# CT imaging findings of epiploic appendagitis: an unusual cause of abdominal pain

**DOI:** 10.1186/s13244-019-0715-9

**Published:** 2019-02-22

**Authors:** Dario Giambelluca, Roberto Cannella, Giovanni Caruana, Leonardo Salvaggio, Emanuele Grassedonio, Massimo Galia, Massimo Midiri, Giuseppe Salvaggio

**Affiliations:** Section of Radiological Sciences, University of Palermo, Via del Vespro 127, 90127 Palermo, Italy

**Keywords:** Adipose tissue, Epiploic appendices, Abdominal pain, Acute abdomen, Differential diagnosis, Large intestine

## Abstract

Epiploic appendagitis is a rare cause of acute abdominal pain, determined by a benign self-limiting inflammation of the epiploic appendages. It may manifest with heterogeneous clinical presentations, mimicking other more severe entities responsible of acute abdominal pain, such as acute diverticulitis or appendicitis. Given its importance as clinical mimicker, imaging plays a crucial role to avoid inaccurate diagnosis that may lead to unnecessary hospitalization, antibiotic therapy, and surgery. CT represents the gold standard technique for the evaluation of patients with indeterminate acute abdominal pain. Imaging findings include the presence of an oval lesion with fat-attenuation surrounded by a thin hyperdense rim on CT (“hyperattenuating ring sign”) abutting anteriorly the large bowel, usually associated with inflammation of the adjacent mesentery. A central high-attenuation focus within the fatty lesion (“central dot sign”) can sometimes be observed and is indicative of a central thrombosed vein within the inflamed epiploic appendage. Rarely, epiploic appendagitis may be located within a hernia sac or attached to the vermiform appendix. Chronically infarcted epiploic appendage may detach, appearing as an intraperitoneal loose calcified body in the abdominal cavity. In this review, we aim to provide an overview of the clinical presentation and key imaging features that may help the radiologist to make an accurate diagnosis and guide the clinical management of those patients.

## Key points

- Epiploic appendagitis is a rare entity causing acute abdominal pain, due to a benign, self-limited inflammation of the epiploic appendages.
- Epiploic appendagitis is a clinical mimicker of other acute abdomen causes, including acute diverticulitis and appendicitis.
- Imaging features of epiploic appendagitis include fat-density ovoid lesion, “hyperattenuating ring sign,” mild bowel wall thickening, and “central dot sign.”

## Introduction

Acute epiploic appendagitis is a rare and easily misdiagnosed cause of acute abdominal pain. It is determined by a benign, self-limiting inflammatory or ischemic damage of the epiploic appendages, or it may be secondary to other inflammatory conditions affecting adjacent abdominal organs. The diagnosis of epiploic appendagitis has a clear clinical relevance and significant implications in patients’ management. In case of acute abdominal pain, this entity is considered a clinical mimicker of other conditions, such as acute appendicitis and diverticulitis, that usually require a surgical evaluation. Imaging plays an essential role in the diagnosis of epiploic appendagitis, allowing the appropriate detection and differential diagnosis, in order to avoid relevant misdiagnosis.

The purpose of this review is to provide an illustrative summary of the normal appearance of epiploic appendages, as well as clinical presentation, with emphasis on most relevant CT findings of epiploic appendagitis and differential diagnosis on imaging.

### Normal epiploic appendages

Epiploic appendages, otherwise known as epiploicae appendices, are pedunculated, adipose outpouchings abutting the serosal surface of the large bowel into peritoneal cavity [1]. There are approximately 50–100 epiploic appendages in adults [2], which are arranged in two separate longitudinal lines, extending from the cecum to the recto-sigmoid junction, located anteriorly along the taenia libera and postero-laterally along the taenia omentalis [3]. The transverse colon harbors a single row, since at this level the taenia omentalis lies at the attachment of the greater omentum. Epiploic appendages can be found also near the vermiform appendix, while they are absent in the rectum.

Normal epiploic appendages are covered by visceral peritoneum and typically measure 1–2 cm in thickness and 2–5 cm in length, but they have been reported to be up to 10 cm in length [4, 5]. For unclear reasons, these structures tend to be larger in obese patients and in those who have recently lost weight [5]. The vascular supply of epiploic appendage relies on one or two small feeding arteries originating from the vasa recta longa of colon, while the venous drainage is guaranteed by a tortuous vein passing through a narrow pedicle [1, 5]. The limited vascular perfusion, associated with their pedunculated shape and increased mobility, exposes epiploic appendages to high risk of torsion with subsequent ischemia or hemorrhage [2–5]. Although the exact role of epiploic appendages is not well understood, it is assumed that these structures act as protective fat pad during intestinal peristalsis, similarly to the greater omentum. They may also play a role in fat storage and intestinal immunity [5].

**Fig. 1:**
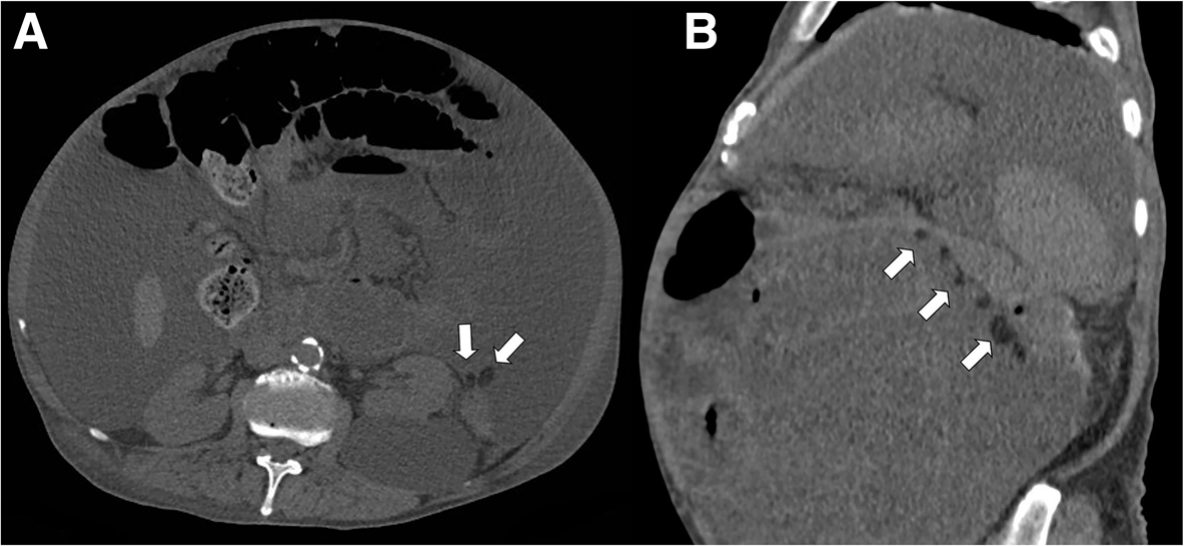
Axial (**a**) and sagittal reformatted (**b**) non-contrast CT images demonstrates massive ascites revealing the normal epiploic appendages (arrows), abutting the splenic flexure of the colon

The normal epiploic appendages are undetectable on computed tomography (CT) images due to the overlapping in density with the surrounding peritoneal and omental fat, while they can be noted when they are inflamed and/or outlined by ascites (Fig. 1) [1].

### Epiploic appendagitis

Epiploic appendagitis, also known as “appendicitis epiploica” or “appendagitis,” is a relatively unusual cause of acute pain in the abdomen, determined by a benign, self-limiting inflammatory or ischemic process affecting the epiploic appendages [2]. The “primary” form of epiploic appendagitis is characterized by ischemic or hemorrhagic infarction of the epiploic appendages, due to the torsion of their pedicles or to the spontaneous central venous drainage thrombosis. These processes result in vascular occlusion and local inflammation. Epiploic appendagitis may also be secondary to other local inflammatory processes affecting adjacent organs, particularly in case of diverticulitis, appendicitis, pancreatitis, or cholecystitis [2, 6, 7].

### Epidemiology and clinical considerations

The exact incidence of epiploic appendagitis is unknown and probably underestimated. In the prior literature, reported incidence rates were 2–7% in patients with initial clinical suspicion of acute diverticulitis or appendicitis [3, 8, 9]. Epiploic appendagitis is more frequently diagnosed between the second and the fifth decades of life, with a reported mean age at diagnosis of 44 years (range 12–82 years) and an incidence four times higher in males than females [2, 3, 10–14].

Epiploic appendagitis may occur in any segment of the colon. It affects more commonly the sigmoid (50%), followed by the descending (26%), ascending colon and cecum (22%), while it is uncommonly encountered in the transverse colon (2%) [10, 11, 13–17]. Obesity and rapid weight loss are described as potential risk factors for the development of epiploic appendagitis [3, 14, 16–18].

The clinical diagnosis of epiploic appendagitis is challenging due to the lack of pathognomonic clinical signs or laboratory markers. Clinical presentation may vary among patients. This condition is often an underestimated and overlooked mimicker, easily misdiagnosed with other causes of abdominal pain requiring urgent care or surgical evaluation in emergency department, particularly acute diverticulitis (left iliac pain) or acute appendicitis (right iliac pain) [10, 12, 19]. On physical examination, patients complain of focal non-migratory abdominal pain in the lower abdominal quadrants (left more commonly than right), not exacerbated by physical movement, without significant guarding or rigidity, and in the absence of a palpable mass [10, 11, 18]. The pain is typically circumscribed and the patient may often point his finger on the place where the lesion is situated. Patients may also report postprandial fullness, swelling, vomiting, early satiety, diarrhea, and rarely mild fever [10, 12, 14, 18, 20, 21]. White blood cell count, erythrocyte sedimentation rate, and C-reactive protein levels are within normal range in the majority of patients, while mild leukocytosis may occasionally be observed due to inflammatory response [10–12, 14, 20, 22].

Epiploic appendagitis is a benign, self-limiting condition which spontaneously resolves within 2 weeks from the onset of symptoms [20, 23]. Surgery is not indicated, since the treatment is conservative with nonsteroidal anti-inflammatory drugs, according to the patient’s symptoms. The risk of recurrence is low and complications are even rarer [19, 22]. Some authors believed that surgical intervention with ligation and excision of the inflamed epiploic appendages was the only way to prevent recurrences and complications, such as adhesions and intussusception induced by inflammation [14]. However, invasive intervention is usually avoided due to the potential complications present in all surgical procedures. In some cases, epiploic appendagitis has been reported as an unexpected finding in patients who underwent laparotomy for acute abdominal pain with clinical suspicion of acute appendicitis or acute diverticulitis [14, 24]. It can also be a diagnosis of exclusion when other causes of acute abdominal pain are disproved.

**Fig. 2:**
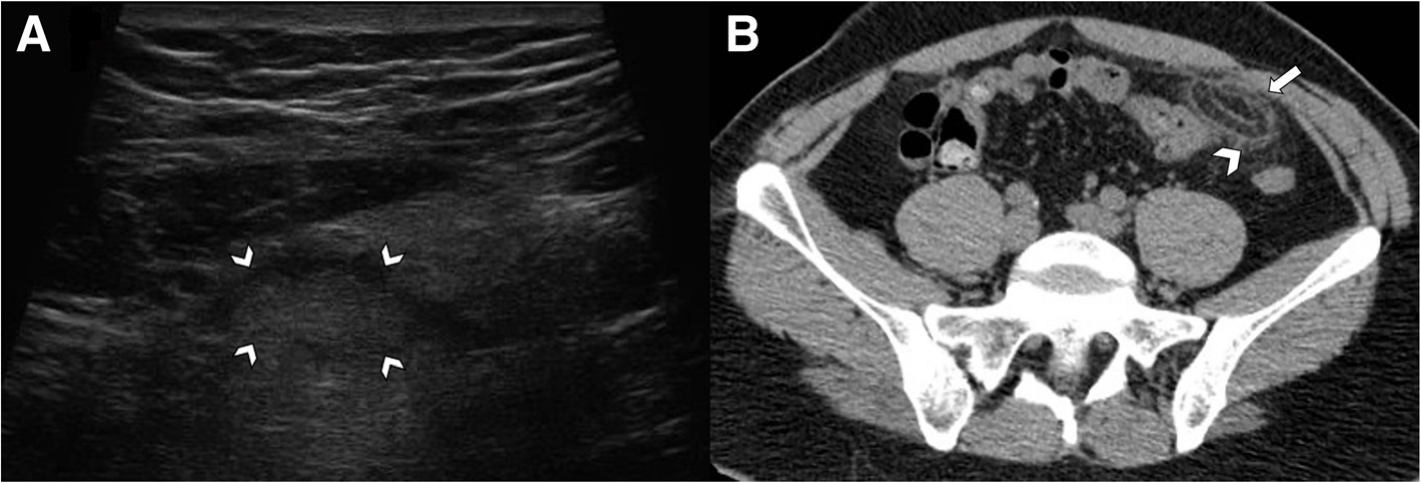
Acute epiploic appendagitis in a 41-year-old man. **a** Ultrasound transverse scan, at the point of maximum tenderness, shows a well-defined hyperechoic ovoid fat lesion, surrounded by hypoechoic rim (arrowheads), adjacent to the anterior abdominal wall. **b** Axial non-contrast CT image shows an inflamed fatty ovoid lesion near the sigmoid colon with hyperattenuating ring (arrow) and nearby fat stranding (arrowhead)

Albeit abdominal ultrasound imaging has the benefit of being guided by the patients’ point of maximum tenderness, it could show no abnormalities in some cases [14]. Thus, CT is nowadays considered the gold standard imaging technique for a definite diagnosis of primary epiploic appendagitis [8]. On ultrasound images, epiploic appendagitis has been described as a hyperechoic incompressible lesion encircled by a subtle hypoechoic rim (Fig. 2a) [8, 10, 22, 25, 26]. Absence of flow on color Doppler images is characteristic of epiploic appendagitis, as opposed to hypervascularization occurring in acute diverticulitis [8, 27]. Although contrast-enhanced CT is the technique of choice for the differential diagnosis of acute abdominal pain, a single non-contrast-enhanced phase is sufficient to diagnose the presence of an inflamed epiploic appendage and, above all, to differentiate this entity from other causes of acute abdomen, particularly acute appendicitis or diverticulitis [23]. MR imaging may be considered to confirm the diagnosis in pediatric population [28].

### CT imaging findings

**Fig. 3:**
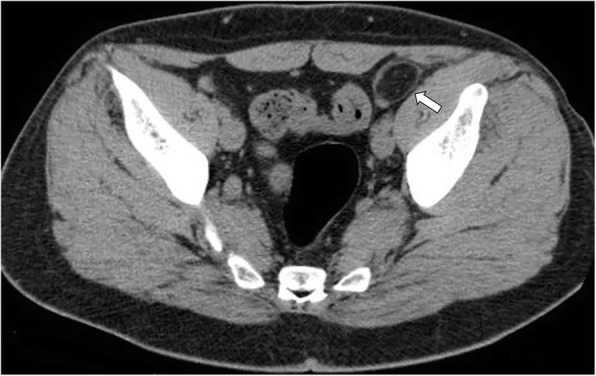
Acute epiploic appendagitis in a 43-year-old man with presumptive clinical diagnosis of colonic diverticulitis. Axial non-contrast CT image shows a pericolonic fat-density lesion (arrow) that abuts anteriorly the colon-sigmoid junction in the left iliac fossa

**Fig. 4:**
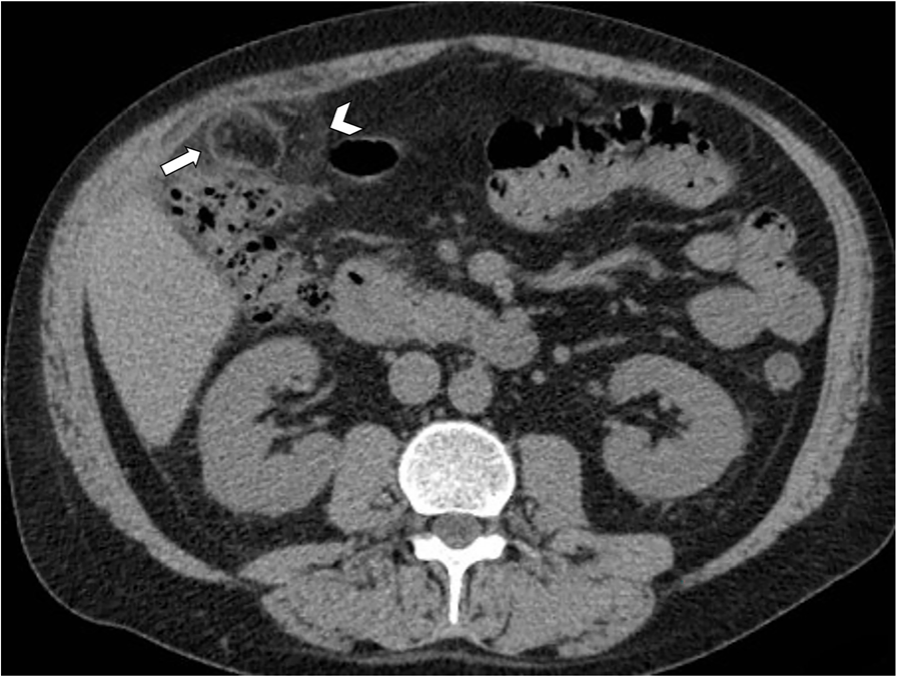
Acute epiploic appendagitis in a 53-year-old man. Axial non-contrast CT image demonstrates a fat-density lesion (arrow) that abuts the hepatic flexure of the transverse colon. Mild fat stranding is surrounding the lesion (arrowhead)

**Fig. 5:**
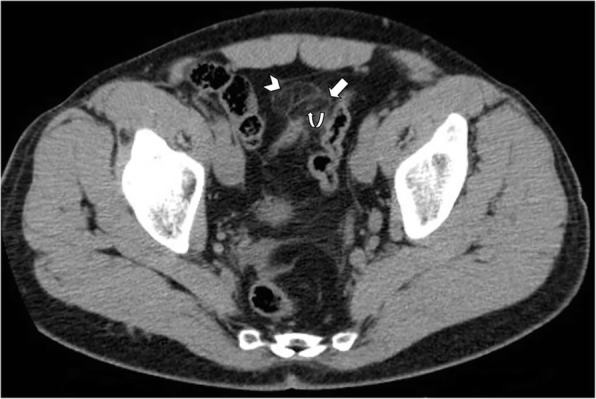
Acute epiploic appendagitis in a 40-year-old man. Axial non-contrast CT image demonstrates a fatty ovoid lesion with high density rim (arrow) that abuts the sigmoid colon and contain central focal area of hyperattenuation (curved arrow), consistent with the “central dot sign” suggestive of thrombosed vascular pedicle. Note also surrounding mild fat inflammation (arrowhead)

CT imaging allows an accurate and non-invasive diagnosis of epiploic appendagitis in most cases [6]. The imaging expression of an inflamed or infarcted epiploic appendagitis on unenhanced CT images is a solitary fat-density ovoid lesion, measuring usually 1–5 cm in axial diameter, abutting anteriorly the large bowel wall (Fig. 3) [11, 13, 14, 16, 29, 30]. A peripheral high attenuation rim (2–3 mm thick) surrounding the ovoid lesion is the expression of the inflamed visceral peritoneum covering the epiploic appendage. The “hyperattenuating ring sign” is a highly suggestive radiological feature of primary epiploic appendagitis and it is used as the primary criterion for diagnosis (Fig. 2b) [16, 19, 23]. Thickening of the parietal peritoneum has also been described as expression of spread of inflammation in about 30–40% of patients [2, 11, 16, 19]. Segmental asymmetric thickening of adjacent colonic wall is usually minimal and disproportionate to the more severe amount of adjacent mesentery inflammation, which appears at imaging with a characteristic “fat stranding” sign (Fig. 4) [2, 4]. This disproportion reflects the primary site of the inflammatory process, located in the pericolonic appendages rather than the bowel wall. A central high-attenuation focus, also known as the “central dot sign”, is highly evocative of central venous pedicle thrombosis within an inflamed epiploic appendage (Fig. 5). Despite this is a pathognomonic imaging feature, it has been variably reported, ranging from 30 to 78% of cases [11–13, 15, 16, 22, 25]; so, even if this sign is absent, a diagnosis of epiploic appendagitis should not be precluded [2, 31]. No enhancement is noted in the epiploic appendagitis or adjacent peritoneum on post-contrast CT images.

**Fig. 6:**
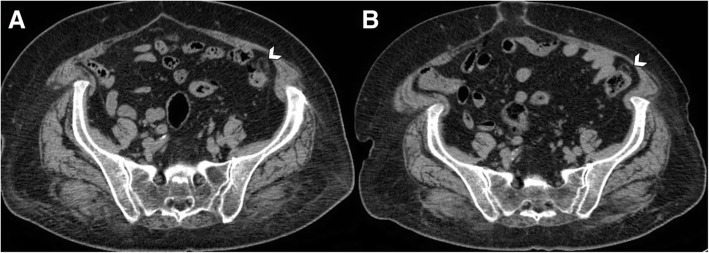
Evolution of acute epiploic appendagitis in a 72-year-old woman. **a** Axial non-contrast CT image shows a pericolonic fat-density lesion (arrowhead) that abuts anteriorly the colon-sigmoid junction in the left iliac fossa. **b** At 3-month follow-up, CT image demonstrates reduction in size of the lesion (arrowhead)

**Fig. 7:**
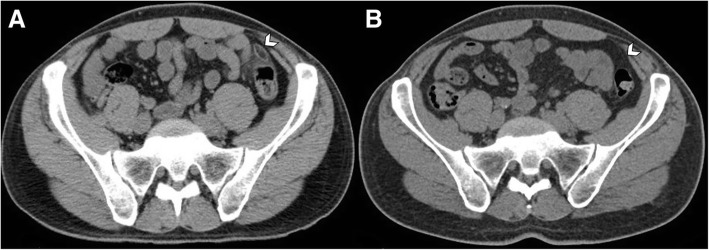
Evolution of acute epiploic appendagitis in a 37-year-old man. **a** Axial non-contrast CT image shows an inflamed fatty ovoid lesion near the sigmoid colon (arrowhead), with hyperattenuating ring sign and nearby fat stranding. **b** CT scan, performed 1 year later for other reasons, demonstrates complete resolution of imaging findings of epiploic appendagitis (arrowhead)

**Fig. 8:**
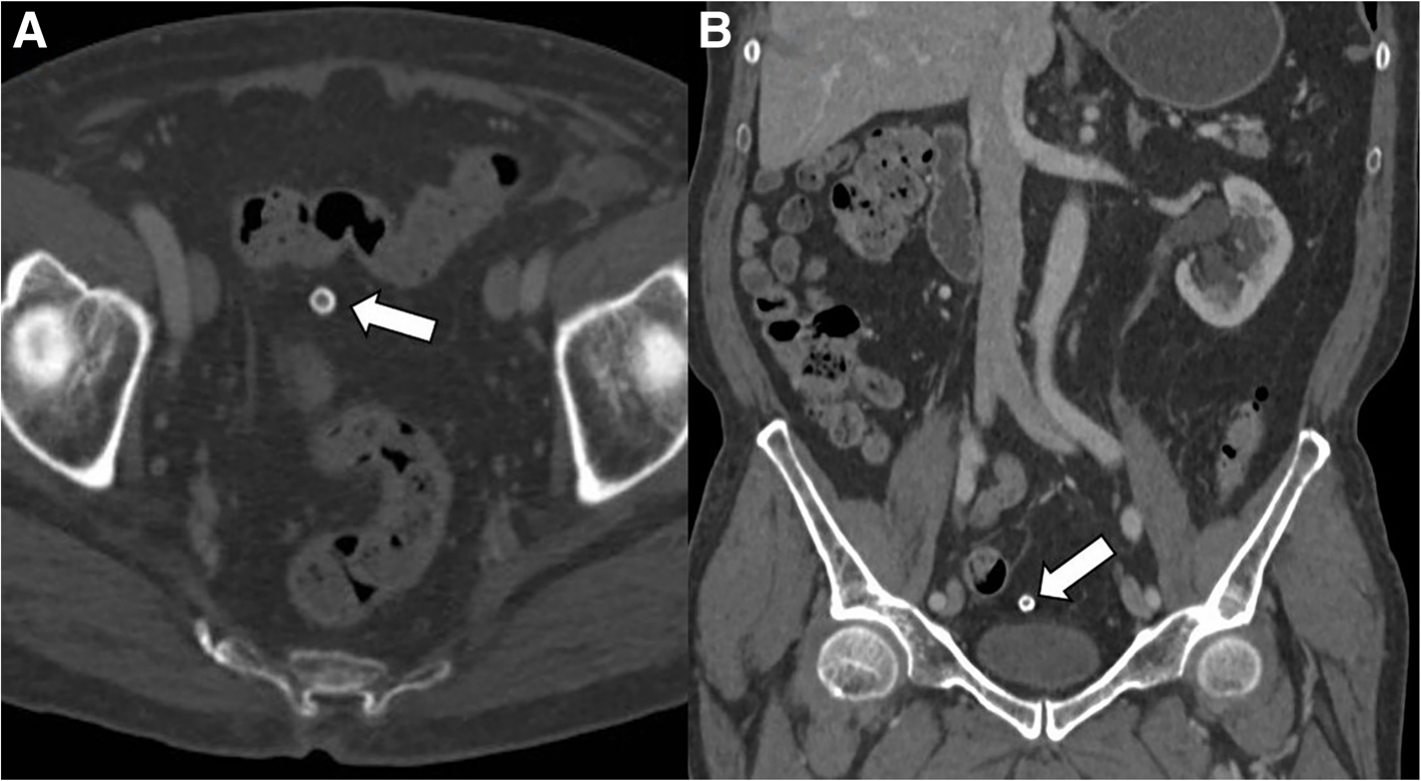
Chronic calcified, amputated epiploic appendage in an 81-year-old man. **a** Axial contrast-enhanced CT image (bone window) shows a calcified, ring-like lesion (arrow) medial to the sigmoid colon which could be mistaken for a mesenteric lymph node or drop gallstone. **b** Coronal reformatted CT image of the same patient depicts the peritoneal loose body in pelvic cavity, likely due to chronic infarcted epiploic appendage (arrow)

The CT imaging findings usually resolve within 6 months after the onset of acute epiploic appendagitis with size reduction or complete regression of the process (Figs. 6 and 7) [1, 32]. Chronically infarcted epiploic appendage may develop aseptic fat necrosis, gradually transforming into a fibrotic or calcified nodule [21]. It may stay attached to the colonic serosa or detach from this latter, appearing as a small, mobile calcified structure within the dependent peritoneal recesses (Fig. 8) [15]. Many of these residual calcified appendages are incidentally found during abdominal surgical procedures, autopsy, or CT scan performed for unrelated conditions. Most of amputated epiploic appendices are small in size (diameter of 1–3 cm) and they are characterized by an oval shape with patchy “popcorn” or peripheral “eggshell” calcification pattern [5, 15]. They can be potentially mistaken at imaging for drop gallstones, calcified lymph nodes, or uterine fibroids [5].

### Atypical imaging

**Fig. 9:**
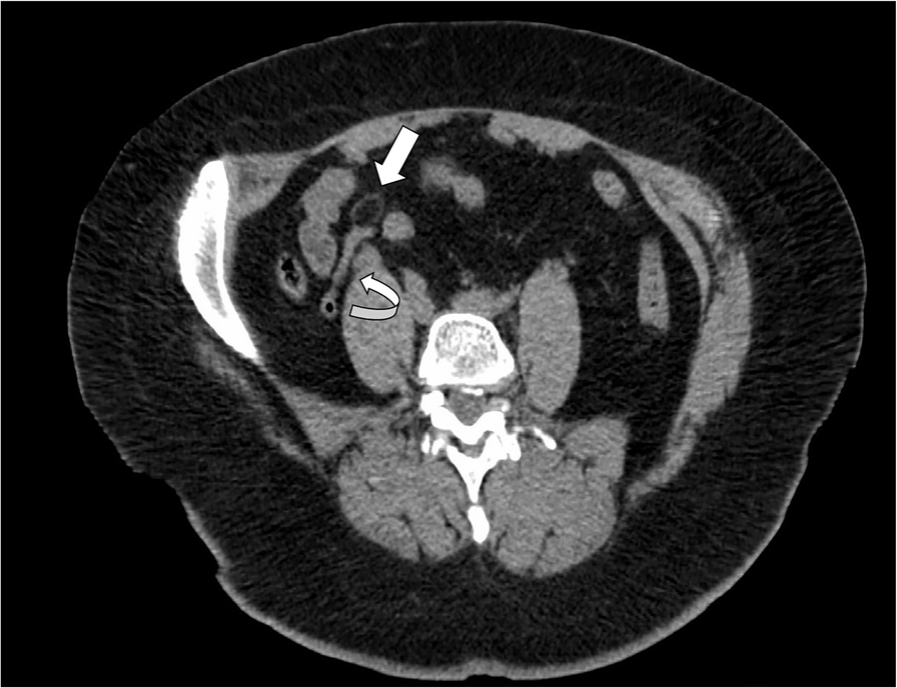
Acute epiploic appendagitis adjacent to the vermiform appendix in a 39-year-old woman. Curved reformatted non-contrast CT image shows an oval lesion with fat attenuation and peripheral hyperdense rim (arrow), that is depending directly from the vermiform appendix tip (curved arrow)

An atypical location of epiploic appendages is near the vermiform appendix. In this case, they have smaller size compared to those adherent to the colonic wall. Epiploic appendagitis of the vermiform appendix is particularly rare and only a limited number of cases have been described in the prior literature (Fig. 9) [33–35]. It is important to distinguish this condition from an acute appendicitis, particularly from tip appendicitis, to avoid unnecessary surgery [35].

**Fig. 10:**
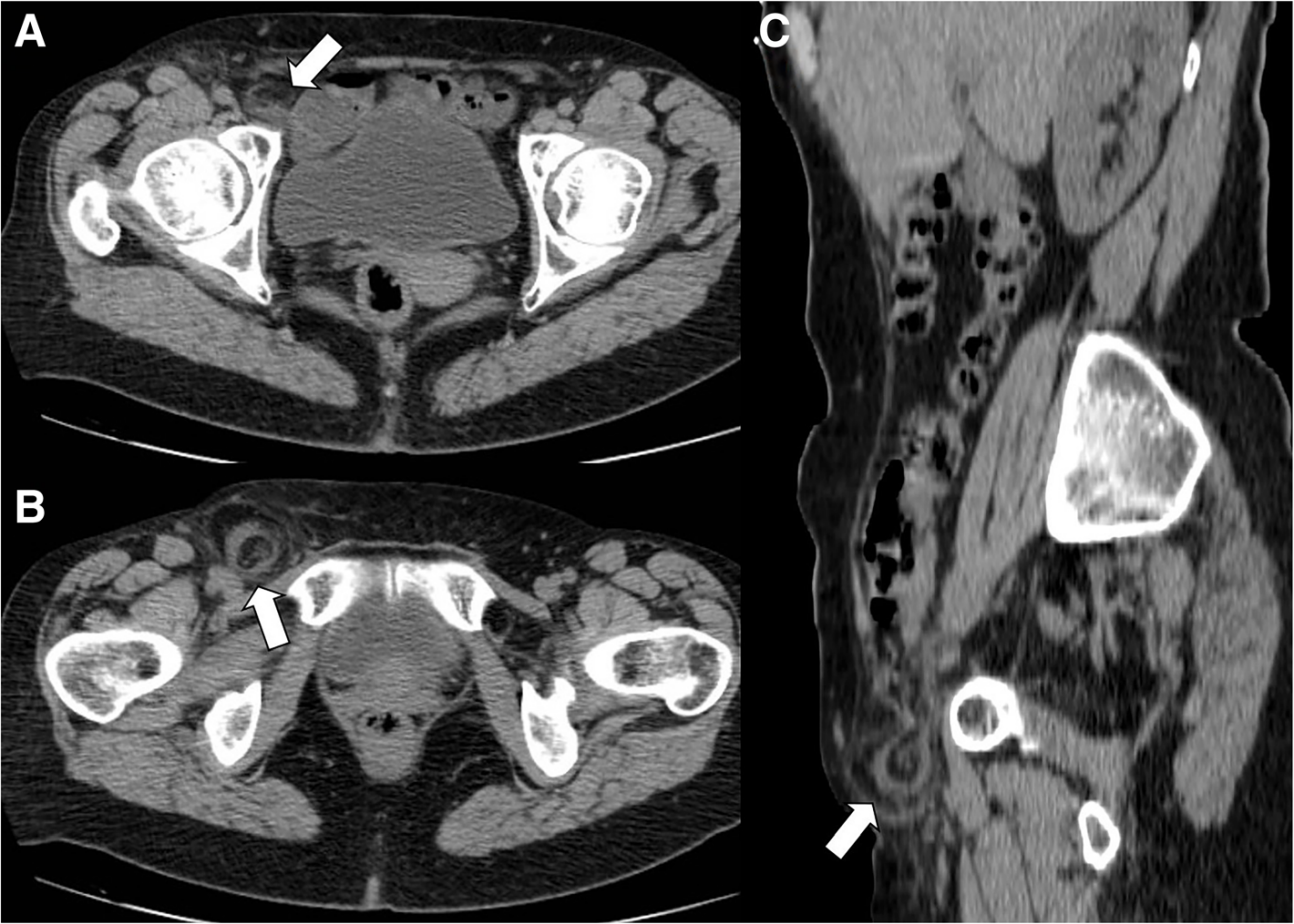
Acute epiploic appendagitis in a 59-year-old woman. Axial non-contrast CT images at two different levels (**a**, **b**) and sagittal reformatted image (**c**) show a right inguinal hernia containing an epiploic appendage surrounded by a thin hyperdense rim (arrows). Fat stranding is seen within the hernia sac. Surgical hernia repair and pathological examination confirmed the presence of an epiploic appendagitis incarcerated in inguinal hernia

In very rare circumstances, an epiploic appendix can protrude into a hernia sac and become an irreducible hernia (Fig. 10) [36–38]. The diagnosis of incarcerated or strangulated hernia, clinically suspected in patients with non-reducible and painful inguinal mass, should be excluded by CT imaging in the absence of radiological signs of intestinal obstruction. However, in these cases, an emergency exploration of the inguinal canal cannot be postponed in order to rule out a possible incarceration of intestinal loops. Treatment involves the excision of epiploic appendix followed by hernia repair (herniorrhaphy or hernioplasty) [36, 39, 40].

Complications of primary epiploic appendagitis have been occasionally reported. The inflammatory process may rarely cause adhesions and lead to small bowel obstruction and thus required surgical treatment [41, 42]. Epiploic appendages are not in continuity with the fecal content, indeed pyogenic abscesses are not a typical complication [43]. In case of CT evidence of intra-abdominal abscess, the diagnosis of epiploic appendagitis secondary to complicated diverticulitis should be considered.

### Differential diagnosis

**Fig. 11:**
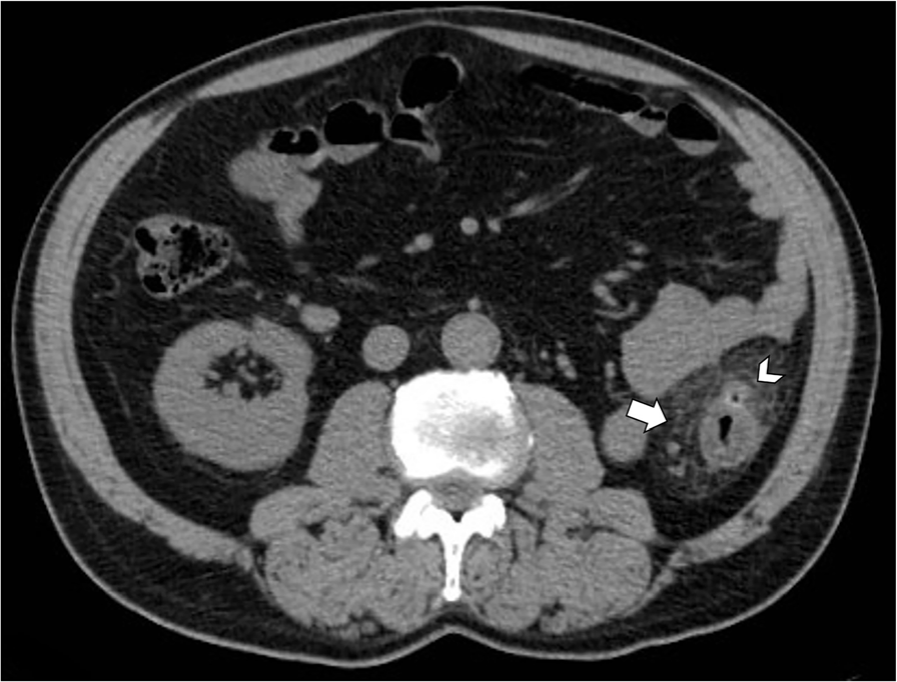
Acute diverticulitis in a 55-year-old man. Axial non-contrast CT image shows segmental thickening of descending colon, with pericolonic fat stranding (arrow). An inflamed diverticulum is identified abutting the anterior colonic wall (arrowhead)

Differential diagnosis for epiploic appendagitis should be made with other conditions presenting as acute abdominal pain in the lower abdominal quadrants. The clinical presentation, together with the results of physical examination, usually leads to a suspected diagnosis of acute appendicitis, acute diverticulitis, or omental infarction. In most cases, non-contrast CT can help distinguish between these entities. Typical CT imaging of acute diverticulitis include colonic wall thickening and pericolonic fat stranding in patients with a history of diverticulosis. White blood cells count and inflammatory markers are significantly higher in acute diverticulitis compared to epiploic appendagitis [19, 44]. The thickening and involvement of the colonic wall is usually much greater in acute diverticulitis than the one reported in the epiploic appendagitis (Fig. 11). Moreover, typical complications of diverticulitis (i.e., perforation, abscess, fistulization) are not seen in epiploic appendagitis [2]. Of note, acute diverticulitis is the most common cause of secondary epiploic appendagitis [6, 7]. Multiple lesions, larger size, and diffuse colon wall thickening may suggest the secondary epiploic appendagitis, rather than a primary form [6]. On the other hand, non-inflamed diverticulosis may coexist in patients with epiploic appendagitis and should not prevent its diagnosis when typical signs are present. Thus, inflammatory features of both diverticula and epiploic appendages should be scrutinized when these entities coexist.

**Fig. 12:**
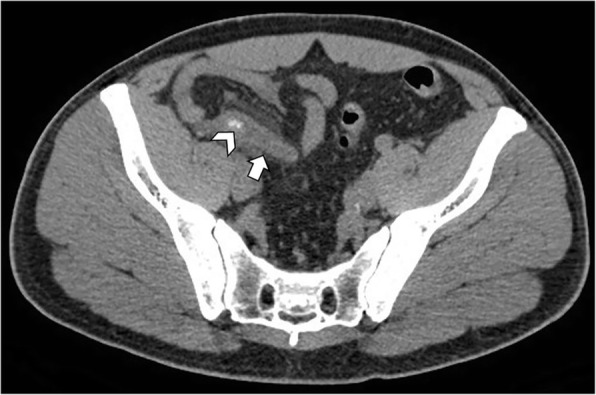
Acute appendicitis in a 43-year-old man. Axial non-contrast CT image shows an inflamed vermiform appendix (arrow), with thickened wall and nearby fat stranding. Appendicoliths are identified within the appendix (arrowhead)

Patients with acute appendicitis present with fever, leukocytosis, nausea, vomiting, and right lower abdominal pain. CT imaging will demonstrate a fluid-filled dilated appendix (> 6 mm) with thickening of the appendicular walls (> 2 mm), hyperdensity of the peri-appendicular fat, and thickening of the adjacent cecal apex [2, 4]. Intraluminal appendicoliths may also be visualized (Fig. 12).

**Fig. 13:**
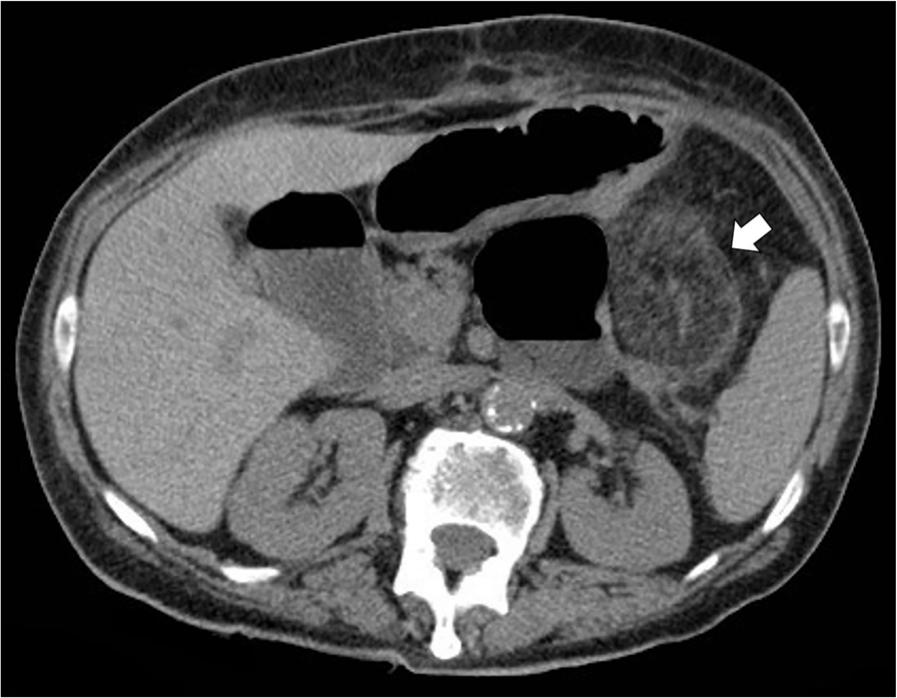
Acute omental infarction in a 67-year-old woman with previous history of total colectomy. Axial non-contrast CT image shows a 6.0 cm heterogeneous fatty ovoid lesion in the left upper quadrant (arrow), associated with inflammatory changes in the nearby fatty tissue

Although epiploic appendagitis can have a similar appearance to acute omental infarction on CT imaging, this distinction has no significant practical consequences, because both conditions share the same clinical presentation and benign evolution. On CT images, omental infarction is characterized by the presence of heterogeneous ill-defined fat-density lesion larger than 3 cm, without the hyperattenuating ring sign observed in epiploic appendagitis (Fig. 13) [2, 32, 45, 46]. Furthermore, omental infarction is located in atypical position, more frequently centered in the omentum or in the right iliac fossa, and not abutting the colon surface as opposed to epiploic appendagitis [4, 45].

Other entities that should be considered in the differential diagnosis are mesenteric panniculitis, acute pancreatitis, fat-containing tumor (i.e., liposarcoma, exophytic angiomyolipoma), or inflammatory pelvic diseases [1, 47].

## Conclusions

Epiploic appendagitis is an undervalued cause of acute abdominal pain, with an ambiguous clinical presentation that often requires evaluation of CT imaging. It is often diagnosed in patients with clinical suspicion of acute appendicitis or diverticulitis. CT should be used to exclude the presence of other more common and relevant causes of abdominal pain and to confirm the benign nature of the lesion. It is strongly recommended to correlate the various imaging characteristics with clinical manifestations, to identify the subgroup of patients who will benefit from possible conservative treatment options using analgesics and to prevent unnecessary hospitalization, antibiotic therapies and surgery.
